# Neural Responsivity to Food Cues in Patients With Unmedicated First-Episode Psychosis

**DOI:** 10.1001/jamanetworkopen.2018.6893

**Published:** 2019-01-11

**Authors:** Faith Borgan, Owen O’Daly, Karen Hoang, Mattia Veronese, Dominic Withers, Rachel Batterham, Oliver Howes

**Affiliations:** 1 Department of Psychosis Studies, Institute of Psychiatry, Psychology and Neuroscience, King’s College London, London, United Kingdom; 2 Psychiatric Imaging Group, Faculty of Medicine, Medical Research Council London Institute of Medical Sciences, Imperial College London, London, United Kingdom; 3 Centre for Neuroimaging Sciences, Institute of Psychiatry, Psychology and Neuroscience, King’s College London, London, United Kingdom; 4 Metabolic Signalling Group, Medical Research Council London Institute of Medical Sciences, Imperial College London, London, United Kingdom; 5 Institute of Clinical Sciences, Imperial College London, London, United Kingdom; 6 Centre for Obesity Research, University College London, London, United Kingdom; 7 University College London Hospitals Bariatric Centre for Weight Management and Metabolic Surgery, London, United Kingdom; 8 National Institute of Health Research University College London Hospitals Biomedical Research Centre, London, United Kingdom

## Abstract

**Importance:**

Schizophrenia is associated with a reduced life expectancy of 15 to 20 years owing to a high prevalence of cardiometabolic disorders. Obesity, a key risk factor for the development of cardiometabolic alterations, is more prevalent in individuals with schizophrenia. Although obesity is linked to the altered reward processing of food cues, no studies have investigated this link in schizophrenia without the confounds of antipsychotics and illness chronicity.

**Objective:**

To investigate neural responsivity to food cues in first-episode psychosis without the confounds of antipsychotic medication or illness chronicity.

**Design, Setting, and Participants:**

A case-control study was conducted from January 31, 2015, to September 30, 2018, in London, United Kingdom, of 29 patients with first-episode psychosis who were not taking antipsychotic medication and 28 matched controls.

**Main Outcomes and Measures:**

Participants completed a food cue paradigm while undergoing a functional magnetic resonance imaging scan. Neural activation was indexed using the blood oxygen level–dependent hemodynamic response. The Dietary Instrument for Nutrition Education was used to measure diet, and the International Physical Activity Questionnaire was used to measure exercise.

**Results:**

There were no significant differences in age, sex, or body mass index between the 29 patients (25 men and 4 women; mean [SD] age, 26.1 [4.8] years) and 28 controls (22 men and 6 women; mean [SD] age, 26.4 [5.5] years). Relative to controls, patients consumed more saturated fat (*t*_46_ = –3.046; *P* = .004) and undertook less high-intensity (*U* = 304.0; *P* = .01) and low-intensity (*U* = 299.5; *P* = .005) weekly exercise. There were no group differences in neural responses to food vs nonfood cues in whole-brain or region-of-interest analyses of the nucleus accumbens, insula, or hypothalamus. Body mass index was inversely correlated with the mean blood oxygen level–dependent signal in the nucleus accumbens in response to food vs nonfood cues in controls (*R* = –0.499; *P* = .01) but not patients (*R* = 0.082; *P* = .70).

**Conclusions and Relevance:**

Relative to controls, patients with first-episode psychosis who were not taking antipsychotic medication consumed more saturated fat and showed an altered association between body mass index and neural response to food cues in the absence of differences in neural responses to food cues. These findings highlight how maladaptive eating patterns and alterations in the association between body mass index and neural responses to food cues are established early in the course of schizophrenia.

## Key Points

**Question:**

Do patients with first-episode psychosis who are not taking antipsychotic medication show altered neural responses to food cues?

**Findings:**

In a case-control study comparing 29 patients with first-episode psychosis who were not taking antipsychotic medication and 28 healthy controls, patients with first-episode psychosis consumed significantly greater amounts of saturated fat relative to healthy controls in the absence of significant differences in neural responses to food cues.

**Meaning:**

Although neural responses to food cues are not intrinsically dysregulated in schizophrenia, maladaptive eating patterns and alterations in the association between body mass index and neural responses to food cues are established early in the course of schizophrenia.

## Teaser

This case-control study investigates neural responsivity to food cues in patients with first-episode psychosis without the confounds of antipsychotic medication or illness chronicity.

## Introduction

Schizophrenia is associated with a reduced life expectancy of 15 to 20 years and greater mortality rates due to a high prevalence of cardiometabolic disorders.^1,2,3^ Obesity, a key risk factor contributing to the development of cardiometabolic dysfunction,^4,5^ is more prevalent among both medicated and unmedicated patients with schizophrenia relative to the general population.^6,7^ Although the mechanisms underlying the development of obesity remain unclear, previous literature has shown that individuals with obesity exhibit altered reward processing in response to food cues.^8,9,10,11^

The hedonic properties of food are processed by the reward system involving the nucleus accumbens, which forms part of the ventral striatum.^12,13,14^ Previous literature has suggested that altered reward processing, potentially mediated by striatal dopamine,^15,16^ may underlie excessive weight gain in individuals with schizophrenia.^17^ In line with this possibility, patients with schizophrenia show altered functional activation during reward processing in the striatum^18,19,20,21^ and an increase in presynaptic dopamine synthesis and release capacity in the striatum.^22,23^

Further evidence for this finding comes from the 2 studies to date that have investigated functional responsivity to food cues in individuals with schizophrenia. Relative to controls, patients with schizophrenia showed decreased striatal activation in response to food cues under fasting conditions^24^ but not under nonfasting conditions.^25^ Under nonfasting conditions, patients with schizophrenia showed greater functional deactivation in the thalamic nucleus, parahippocampal gyrus, and middle frontal lobe and decreased activation in the bilateral inferior parietal lobe.^25^

However, the patients in both these studies were taking antipsychotic medications, which block striatal dopamine signaling^26^ and alter striatal functional activation.^18,27,28^ Thus, it remains unclear whether altered striatal responses to food cues are secondary to the effects of antipsychotics or are an intrinsic component of the pathophysiologic characteristics of the disorder. Determining whether there is a primary alteration in the processing of food cues is critical to guiding the development of interventions to prevent and treat weight gain and cardiometabolic dysfunction in individuals with schizophrenia.

In view of this, we aimed to investigate whether in the fasted state, patients with first-episode psychosis, who were not taking antipsychotic medication, show functional alterations in the ventral striatum in response to food cues. Consistent with the previous study, although it was conducted in patients taking antipsychotic treatment,^25^ we hypothesized that patients who were not taking antipsychotic medication would show decreased functional activation in the ventral striatum in response to food cues.

## Methods

### Participants

In this case-control study, 57 volunteers, including 29 patients with first-episode psychosis and 28 healthy controls matched by age were recruited in London, United Kingdom, between January 31, 2015, and September 30, 2018. The patients with first-episode psychosis were recruited from early intervention services for psychosis, and the healthy controls were recruited via local advertising. After screening 459 individuals, 35 patients were enrolled in the study. Because 6 of these patients had started antipsychotic treatment, only 29 patients were included in the final analysis. Based on a study investigating striatal activation in response to food vs nonfood cues in individuals with schizophrenia,^24^ albeit in patients with chronic schizophrenia, a power calculation indicated that 9 individuals per group were needed to allow for the detection of effect sizes greater than 0.9 (α = .05) using a 2-tailed hypothesis. The study obtained ethical approvals from the Camberwell St Giles ethics committee and adhered to the guidelines described by the Declaration of Helsinki.^29^ Written informed consent was obtained from all participants. This study followed the Strengthening the Reporting of Observational Studies in Epidemiology (STROBE) reporting guidelines.

### Inclusion and Exclusion Criteria

All participants were 18 to 65 years of age and were able to give written informed consent. Individuals were excluded if they had any of the following: (1) a history of a head injury leading to loss of consciousness; (2) contraindications to undergoing magnetic resonance imaging (MRI); (3) current or lifetime history of substance use or dependence as determined by the Structured Clinical Interview for *DSM-IV-TR* (SCID-I/P); (4) comorbid axis I diagnosis as determined by the *DSM-IV-TR* (SCID-I/P); (5) current or recent (within the last month) recreational use of illicit substances; (6) positive results on a Δ^9^-tetrahydrocannabinol urine toxicology test with a 0.05-μg/mL cutoff that can detect 11-nor-9-carboxy-Δ^9^-tetrahydrocannnabinol metabolites for up to 30 days; (7) positive results on a multipanel drug screening test detecting cocaine, amphetamine, cannabis, opiates, and benzodiazepines; or (8) a history of neurological diseases or other brain abnormalities. Healthy controls met the following inclusion criteria: no current or lifetime history of an axis I disorder as determined by the SCID-I/P and no family history of an axis I disorder in first-degree and second-degree relatives as determined by the Family Interview for Genetic Studies.^30^ There were no exclusion criteria for weight or body mass index (BMI) for all participants. Patients with first-episode psychosis met the following inclusion criteria: diagnosis of schizophrenia or schizoaffective disorder as determined by the SCID-I/P; antipsychotic naive or free from all pharmacological treatments acting on the central nervous system (eg, antipsychotics, antidepressants, benzodiazepines) for at least 6 weeks; no prior use of depot medication; and illness duration of less than 3 years.

### Demographic Characteristics

Age, sex, weight, and height were recorded. Socioeconomic status, employment status, and race/ethnicity were defined by the participant and classified using the Medical Research Council Sociodemographic Schedule.^31^ Educational level and years of education were recorded using the methods described in Table 1. Body mass index was calculated using the methods described elswhere.^32^ Current and previous use of alcohol, nicotine, and illicit substances were recorded.

**Table 1.** Sample Clinical and Demographic Characteristics

Characteristic	Healthy Controls (n = 28)	Patients With First-Episode Psychosis (n = 29)	Statistic	*P* Value
Age, mean (SD), y	26.4 (5.5)	26.1 (4.8)	*t*_55_ = 0.265	.79
Sex, No.				
Male	22	25	χ^2^_1_ = 0.574	.45
Female	6	4		
Race/ethnicity, No.				
White	10	12	χ^2^_3_ = 5.303	.15
Black African or Black Caribbean	3	9		
Asian	11	6		
Mixed	4	2		
Employment, No.				
Full-time	10	3	χ^2^_4_ = 19.455	.001
Part-time	2	7		
Unemployed	2	12		
Student	13	4		
Missing data	1	3		
Education, No.				
Completed high school	3	8	χ^2^_2_ = 3.716	.16
Did not complete high school	8	9		
Completed university	16	10		
Missing data	1	2		
Years of education after compulsory education, median (IQR)^a^	4.0 (2.0-5.0)	2.0 (0.5-5.0)	*U* = 379.5	.049
Socioeconomic status, No.^b^				
High	1	1	χ^2^_4_ = 7.112	.13
Medium	4	3		
Low	9	14		
Unemployed	0	3		
Student	12	5		
Missing data	2	3		
Body mass index, mean (SD)^c^	24.7 (37.7)	25.2 (5.0)	*t*_36_ = −0.395	.70
Intake as measured by DINE, mean (SD), servings of food items/wk				
Saturated fat	23.1 (8.5)	31.5 (11.6)	*t*_46_ = −3.046	.004
Unsaturated fat	8.4 (2.67)	8.2 (2.7)	*t*_46_ = 0.271	.79
Fiber	28.9 (8.5)	33.3 (11.5)	*t*_46_ = −1.515	.14
Weekly exercise as measured by IPAQ, median (IQR), min				
High intensity				
Quantity	3.0 (1.0-4.0)	0.0 (0.0-3.0)	*U* = 304.0	.01
Duration	60.0 (20.0-90.0)	0.0 (0.0-90.0)	*U* = 350.5	.06
Moderate intensity				
Quantity	2.0 (0.0-3.0)	1.0 (0.0-2.0)	*U* = 394.5	.29
Duration	60.0 (0.0-90.0)	0.0 (0.0-60.0)	*U* = 362.0	.13
Low intensity				
Quantity	7.0 (7.0-7.0)	5.0 (1.5-7.0)	*U* = 299.5	.005
Duration	60.0 (30.0-120.0)	30.0 (10.5-80.0)	*U* = 373.5	.13
Current cannabis use, No.				
Yes	0	0	NA	NA
No	28	29		
Current alcohol use, No.				
Yes	16	15	χ^2^_1_ = 0.014	.91
No	12	12		
Missing data	0	2		
Current tobacco use, No.				
Yes	9	12	χ^2^_1_ = 0.881	.35
No	19	15		
Missing	0	2		
Diagnosis, No.				
Schizophrenia	NA	27	NA	NA
Schizoaffective disorder	NA	2		
Illness duration, mean (SD), mo	NA	21.5 (11.9)	NA	NA
Duration of prior treatment, mean (SD), mo	NA	4.74 (6.73)	NA	NA
Current use of antipsychotics, No.				
Yes	NA	0	NA	NA
No	NA	29		
Prior use of antipsychotics, No.				
Yes	NA	20	NA	NA
No	NA	9		
Current use of antidepressants, No.				
Yes	NA	0	NA	NA
No	NA	29		
Prior use of antidepressants, No.				
Yes	NA	8	NA	NA
No	NA	21		
PANSS score, mean (SD)				
Positive	NA	27.2 (15.0)	NA	NA
Negative	NA	24.0 (5.1)	NA	NA
General	NA	42.0 (8.8)	NA	NA
Total	NA	89.3 (17.3)	NA	NA

Abbreviations: DINE, Dietary Instrument for Nutrition Education; IPAQ, International Physical Activity Questionnaire; IQR, interquartile range; NA, not applicable; PANSS, Positive and Negative Syndrome Scale.

^a^ Years of education calculated as the years of education after compulsory education (minimum compulsory education in the United Kingdom is 12 years).

^b^ Socioeconomic status: high = high, intermediate, and lower grade professionals; medium = small employer, self-employed, and lower technical occupations; low = sales, routine occupations, unemployed; and student = student.

^c^ Calculated as weight in kilograms divided by height in meters squared.

### Clinical Assessments

The age at illness onset, illness duration, and clinical symptom severity were determined using the Positive and Negative Syndrome Scale.^33^ Healthy controls were screened for personal and family history of mental health problems using the SCID-I/P and the Family Interview for Genetic Studies.^30^

### Diet and Exercise Assessments

Self-reported questionnaires were used to measure individuals’ weekly dietary intake of saturated fat, unsaturated fat, and fiber using the Dietary Instrument for Nutrition Education.^34^ Weekly exercise levels were recorded using the International Physical Activity Questionnaire.^35^

### Neuroimaging

All participants completed an MRI scan acquired on a General Electric MR750 3.0-T scanner at the Centre for Neuroimaging Sciences at King’s College London. The MRI scans were performed between 9:00 and 11:30 am; participants fasted (water was allowed) for more than 12 hours before undergoing the MRI scan. The food cue functional MRI (fMRI) paradigm as described by Karra and colleagues^36^ was used. During the task, participants viewed 192 color photographs including 64 photographs of high-calorie items, 64 photographs of low-calorie items, and 64 photographs of nonfood items. Images of sweet and savory food items were presented for high-calorie items (eg, lasagna, casserole, and cake) and low-calorie items (eg, vegetables, prawn noodle salads, and fruit). Nonfood items included 64 images of household and office supplies (eAppendix in the Supplement).

High-resolution, 3-dimensional, spoiled gradient recalled T1 images were collected (in-plane matrix size, 256 × 256; field of view, 26.0 mm) with a whole-brain, interleaved bottom-up acquisition using a sagittal orientation and using an 8-channel head coil (repetition time, 7.34 milliseconds; echo time, 3.036 milliseconds; inversion time, 4 seconds; flip angle, 11°; slice thickness, 1.2 mm; slice gap, 1.2 mm; and sequence duration, 14 minutes and 6 seconds). A total of 294 fMRI echo planar imaging 2-dimensional gradient echo volumes were collected (in-plane matrix size, 64 × 64; field of view, 21.1 mm), with a whole-brain, sequential bottom-up acquisition for each functional time point using an oblique orientation and using an 8-channel head coil (repetition time, 2 seconds; echo time, 30 milliseconds; flip angle, 75°; 39 slices per volume; slice thickness, 3 mm; between-slice gap, 0.3 mm; and sequence duration, 14 minutes and 54 seconds).

### fMRI Analysis

Data were analyzed with Statistical Parametric Mapping software (SPM-12; version 6684)^37^ using Matlab, version 8.5.^38^ The preprocessing pipeline consisted of the manual reorientation of the structural and functional images so that the anterior commissure lay on the origin ([0, 0, 0]-coordinate), slice timing correction, head motion correction, coregistration of the functional image to the structural file, segmentation, normalization to Montreal Neurological Institute space, and smoothing using an 8-m Gaussian kernel to minimize noise and residual differences in gyral anatomy. The blood oxygen level–dependent (BOLD) response was modeled using a block design in which a canonical hemodynamic response function was convolved with regressors encoding the onset and duration for the following block conditions: high-calorie cues, low-calorie cues, and nonfood cues. Rest trials were left unmodeled and served as an implicit baseline. Ratings of the subjective appeal of cues were modeled as parametric modulators to control for block-to-block variance in individual subjective responses to food images and thereby reduce residual variance. Framewise displacement was calculated using methods described elsewhere.^39^ High-velocity motion spikes were regressed out by including scan nulling (censoring) regressors for volumes with volume-to-volume framewise displacement greater than 0.5 mm. Individual fixed-effects analyses were performed for each participant to identify regional differences in relative activation using the following linear contrasts of parameter estimates: food (high-calorie + low-calorie) cues vs nonfood cues, high-calorie cues vs low-calorie cues, and high-calorie cues vs nonfood cues.

### Statistical Analysis

Data normality was assessed using the Shapiro-Wilk test. Group differences were determined using the χ^2^ test and the independent samples *t* test for normally distributed categorical variables and the independent samples *t* test for continuous demographic variables. Group differences for variables that were not normally distributed were investigated using the Mann-Whitney test.

Independent samples *t* tests were used to investigate group differences in mean framewise displacement and mean ratings of the subjective appeal of high-calorie, low-calorie, and nonfood cues. Group-level random-effects analyses involved a 1-sample *t* test used to examine the effects of food (high-calorie + low-calorie) cues vs nonfood cues, high-calorie cues vs low-calorie cues, and high-calorie vs nonfood cues in controls. Independent samples *t* tests were used to examine group differences between controls and patients in response to food (high-calorie + low-calorie) cues vs nonfood cues, high-calorie cues vs low-calorie cues, and high-calorie cues vs nonfood cues. Age and mean framewise displacement during the fMRI scan were included as covariates. Independent samples *t* tests were also used to examine group differences between controls and patients in neural response to food (high-calorie + low-calorie) cues relative to nonfood cues. For these models, BMI or saturated fat intake were included as a second-level covariate. In all cases, a result was deemed significant if it survived familywise error correction on the basis of the peak-level extent (*P* < .05) using the Statistical Parametric Mapping default uncorrected height threshold of *P* < .001 for cluster formation.

To test our primary hypothesis that functional activation to food cues would be reduced in patients with first-episode psychosis, region-of-interest (ROI) analyses were conducted for the nucleus accumbens and insula, defined using the Hammersmith Atlas,^40^ in addition to the hypothalamus, defined using the Brodmann atlas and the wfu pickatlas tool (Statistical Parametric Mapping). Independent samples *t* tests were conducted to investigate regional (ROI-based) group differences in response to food cues relative to nonfood cues, high-calorie cues vs low-calorie cues, and high-calorie vs nonfood cues. In all cases, a result was deemed significant if it survived familywise error correction on the basis of the peak-level extent (*P* < .05) using a height threshold of *P* < .001 for cluster formation. For a priori ROI analyses, small volume corrections using familywise error corrections for multiple comparisons on the basis of response amplitude (peak-level) were used (indicated by a corrected *P* < .05). Mean activation in the nucleus accumbens in response to food (high-calorie + low-calorie) cues vs nonfood cues was extracted using Marsbar (Statistical Parametric Mapping) using an independently derived ROI obtained from the Hammersmith Atlas.^40^ Pearson correlations were used to investigate the association between mean striatal activation in response to food vs nonfood cues and BMI, weekly dietary intake of saturated fat, unsaturated fat, and fiber. All *P* values were from 2-tailed tests, and results were deemed statistically significant at *P* < .05.

## Results

### Participants

Fifty-seven fasting volunteers (29 patients [25 men and 4 women]; mean [SD] age, 26.1 [4.8] years; 28 controls [22 men and 6 women]; mean [SD] age, 26.4 [5.5] years) took part in the study. There were no significant group differences in age, sex, weight, BMI, race/ethnicity, educational level, or socioeconomic status (Table 1). However, there were significant group differences in employment (χ^2^_4_ = 19.455; *P* = .001), quantity of weekly high-intensity exercise (*U* = 304.0; *P* = .01), low-intensity exercise (*U* = 299.5; *P* = .005), and weekly dietary intake of saturated fat (*t*_46_ = –3.046; *P* = .004). However, there were no group differences in dietary intake of unsaturated fat (*t*_46_ = 0.271; *P* = .79) or fiber (*t*_46_ = –1.515; *P* = .14) (Table 1). During the food cue fMRI paradigm, there were no group differences in mean framewise displacement (*t*_55_ = –0.752; *P* = .45) or self-reported ratings of the appeal of high-calorie cues (*t*_52_ = 0.060; *P* = .95), low-calorie cues (*t*_52_ = 0.930; *P* = .35), or nonfood cues (*t*_52_ = –0.659; *P* = .51).

### Neuroimaging Results

#### Within-Group Analyses: Healthy Controls

**Figure 1.**
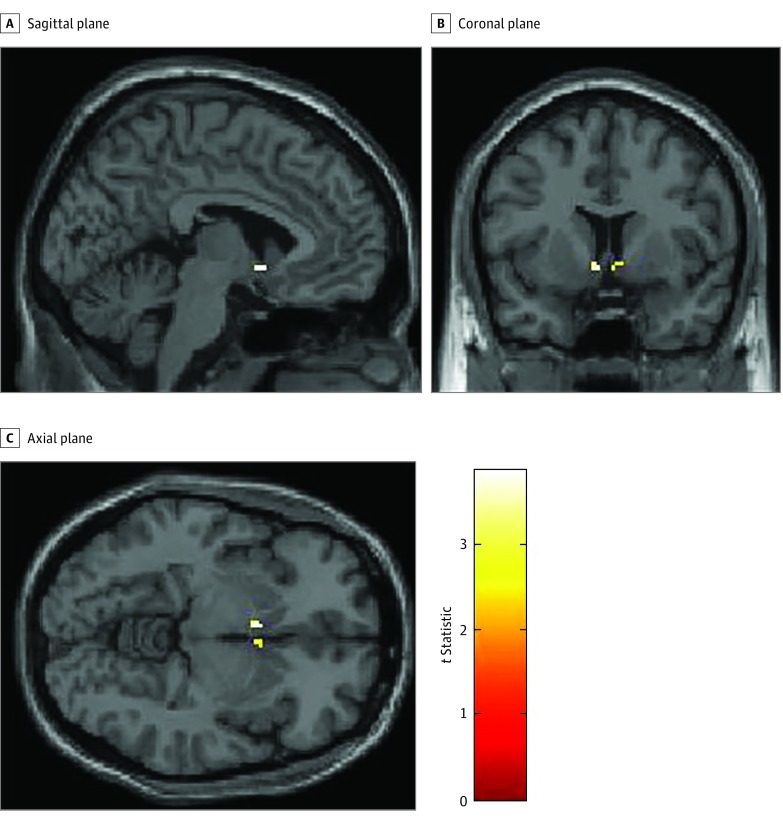
Neural Responses to Food Cues in Healthy Controls Using a Region-of-Interest Analysis of the Nucleus Accumbens Statistical Parametric Mapping *t* maps showing functional activation in the nucleus accumbens in healthy controls in response to food vs nonfood cues. Colors signify relative changes in functional activation (red, low *t* statistic; yellow, high *t* statistic).

**Figure 2.**
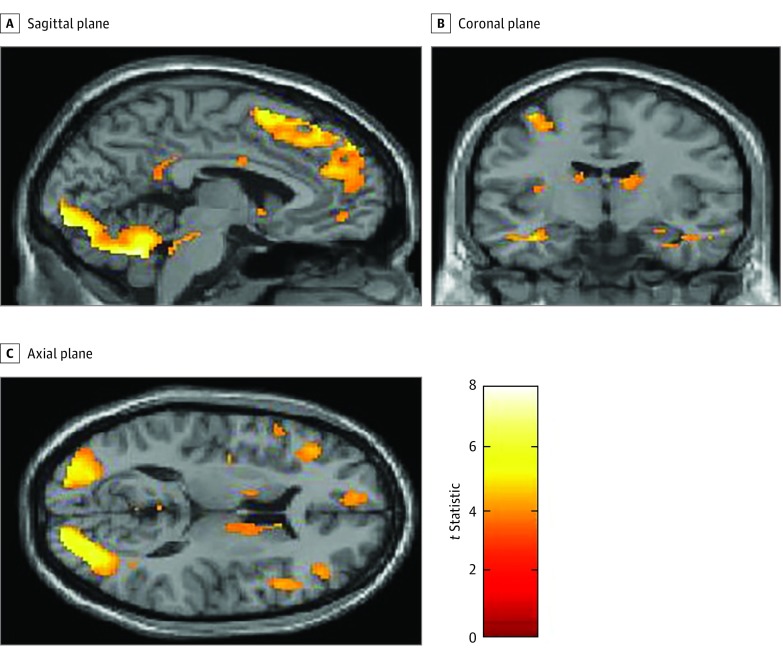
Neural Responses to Food Cues in Healthy Controls Using a Whole-Brain Analysis Statistical Parametric Mapping *t* maps showing functional activation across the whole brain including the insula, nucleus accumbens, and orbitofrontal cortex in healthy controls in response to food vs nonfood cues. Colors signify relative changes in functional activation (red, low *t* statistic; yellow, high *t* statistic).

In a whole-brain analysis, controls showed a greater BOLD signal in response to food cues vs nonfood cues in the right insula and the right anterior, posterior, medial, and inferior orbitofrontal gyrus (Figure 1 and Table 2). In ROI analyses, controls showed a greater BOLD signal in the nucleus accumbens in response to food cues vs nonfood cues (*t* = 3.87; *z* = 3.39; cluster size = 6; *P* = .005 corrected for familywise error; Montreal Neurological Institute coordinates x = –4, y = 8, z = –6) (Figure 2), but not in the insula or hypothalamus. When controls were in a fasting state, they showed no differences in BOLD signal in response to high-calorie cues vs low-calorie cues or high-calorie cues vs nonfood cues in whole-brain analyses or ROI analyses of the nucleus accumbens, insula. or hypothalamus.

**Table 2.** Whole-Brain Analysis Results of the Effects of Food Cues Relative to Nonfood Cues

Group, Cluster	Hemisphere	MNI Coordinates x, y, z	*t* Value	*z* Score	CS, mm^3^	*P* Value^a^
Controls						
Cerebellum	R	44, –64, –34	8.01	5.59	2518	.001
Superior frontal gyrus, middle frontal gyrus	L	–22, 18, 58	7.99	5.58	17	.001
Middle frontal gyrus, precentral gyrus, superior frontal gyrus	L	–34, 2, 58	7.83	5.51	77	.001
Cerebellum	L	–6, –54, –32	7.55	5.40	211	.001
Superior frontal gyrus, superior frontal gyrus medial segment	L	–8, –36, 54	7.41	5.34	61	.002
Occipital pole, calcarine cortex, lingual gyrus, fusiform gyrus, occipital fusiform gyrus, inferior occipital gyrus	L	–10, –98, –2	7.40	5.34	1540	.002
Posterior, lateral, medial, and anterior orbital gyri	R	32, 30, –18	7.13	5.22	34	.002
Superior frontal gyrus, superior frontal gyrus medial segment	R	14, 54, 38	7.01	5.17	43	.003
Controls and patients						
Occipital fusiform gyrus, inferior occipital gyrus, cerebellum	R	38, –74, –16	9.58	7.27	5525	<.001
Occipital fusiform gyrus, inferior occipital gyrus, inferior lingual gyrus	R	–28, –80, –14	9.26	7.11	3449	<.001
Superior frontal gyrus, superior frontal gyrus medial segment, supplementary motor cortex	L	–10, 32, 56	7.08	5.91	45	<.001
Posterior, lateral, anterior, and medial orbital gyri	R	32, 32, –16	6.91	5.81	126	<.001
Middle frontal gyri, precentral gyri	L	–36, 6, 58	6.40	5.48	54	<.001
Superior parietal lobule, angular gyrus	L	–24, –64, 52	6.07	5.26	340	.001
Superior frontal gyrus, middle frontal gyrus	L	–20, 18, 60	5.84	5.11	12	.002
Middle frontal gyrus	R	48, 34, 28	5.77	5.06	90	.002
Precentral gyrus, middle frontal gyrus	L	–52, 8, 40	5.68	4.99	49	.004
Controls > patients^b^						
No suprathreshold clusters	NA	NA	NA	NA	NA	NA
Patients > controls^b^						
No suprathreshold clusters	NA	NA	NA	NA	NA	NA

Abbreviations: CS, cluster size; L, left; MNI, Montreal Neurological Institute; NA, not applicable; R, right.

^a^ *P* value surviving familywise error correction on the basis of peak-level extent using the Statistical Parametric Mapping default using an uncorrected height threshold of *P* < .001 for cluster formation.

^b^ In the comparison of patients and controls, patients did not show greater levels of activation relative to controls, and vice versa.

#### Between-Group Analyses: Healthy Controls vs Patients

**Figure 3.**
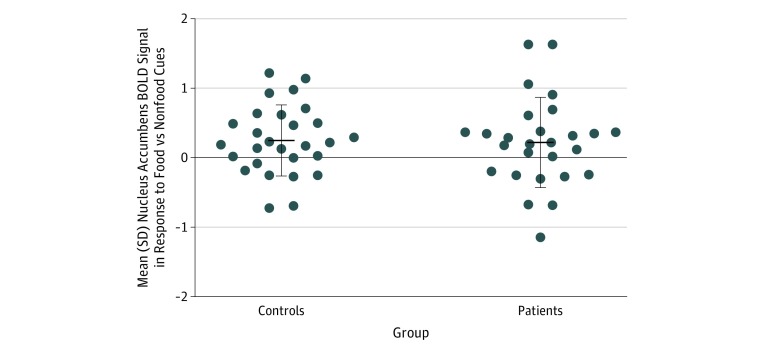
Striatal Activation in Response to Food Cues in Healthy Controls and Patients With First-Episode Psychosis Scatter plot showing the mean and variance of functional activation in the nucleus accumbens in healthy controls and patients with first-episode psychosis in response to food vs nonfood cues. BOLD indicates blood oxygen level–dependent. Error bars indicate SD.

There were no group differences when using contrasts and whole-brain or ROI analyses of the insula, hypothalamus, or the nucleus accumbens of controls and patients for food vs nonfood cues, high-calorie cues vs low-calorie cues, or high-calorie cues vs nonfood cues. Controls did not show greater activation relative to patients, or vice versa. These findings remained unchanged when including BMI or saturated fat intake as covariates. See Figure 3 for the mean values and variance of the BOLD signal in the nucleus accumbens in response to food images vs nonfood images in patients with first-episode psychosis and controls.

#### Association Between Neural Responses to Food Cues, BMI, and Diet

Controls showed a significant inverse association between BMI and mean activation in the nucleus accumbens in response to food cues vs nonfood cues (*R* = –0.499; *P* = .01), but patients did not (*R* = 0.082; *P* = .70); a Fisher *r*-to-*z* transformation indicated that associations were significantly different between groups (*z* = –2.25; *P* = .02 [2-tailed]) (eFigures 1 and 2 in the Supplement). Controls showed no association between mean activation in the nucleus accumbens in response to food cues vs nonfood cues and dietary intake of saturated fat (*R* = –0.103; *P* = .63), unsaturated fat (*R* = –0.040, *P* = .85), or fiber (*R* = –0.232; *P* = .28). Patients showed no association between mean activation in the nucleus accumbens in response to food cues vs nonfood cues and weekly dietary intake of saturated fat (*R* = 0.158; *P* = .48), unsaturated fat (*R* = 403; *P* = .06), or fiber (*R* = 0.158, *P* = .48).

## Discussion

Our main finding is that patients with first-episode psychosis show normal neural responses to food cues relative to controls in the fasted state. Our findings are consistent with those of a previous study reporting no differences in striatal functional activation in response to food cues vs nonfood cues between controls and patients with chronic schizophrenia who were taking antipsychotics,^25^ but they are at odds with the findings from a study reporting greater deactivation in the striatum in patients with chronic schizophrenia who were taking antipsychotics.^24^ Although both studies investigated patients with chronic schizophrenia who were taking antipsychotics, these studies used different fasting protocols, paradigms, statistical thresholds, and corrections for multiple comparisons. However, our finding that patients with first-episode psychosis consume greater levels of saturated fat is consistent with previous literature showing that patients with chronic schizophrenia who are taking antipsychotics consume diets higher in saturated fat compared with healthy controls.^41,42,43^

Our study extends these prior findings in 3 important ways. First, by studying patients with first-episode psychosis, we show that the neural processing of food cues is unaltered early in the illness. Second, by studying patients who are not taking any pharmacological compounds acting on the central nervous system, including medication or illicit substances, we show, for the first time to our knowledge, that patients not taking antipsychotic medication show no evidence of altered neural processing of food cues. Third, by using a fasting protocol, in contrast to the nonfasting protocol used in a previous study of patients with chronic schizophrenia,^25^ we avoid the potential confound of differing states of satiation.

### Strengths and Limitations

A strength of this study was that all participants had not been taking any compounds acting on the central nervous system, including pharmacological treatments or illicit substances. Although glucose levels were not measured, participants completed a fast for a minimum of 12 hours during which they were not permitted to eat or drink anything other than water. As such, the consumption of food or sugary drinks is unlikely to be a confound but cannot be excluded. Although many patients were antipsychotic naive, some patients not taking antipsychotic medication had taken antipsychotic medication in the past. However, this is unlikely to influence the results because the patients had a minimum drug washout period of 6 weeks and no patients had previously received depot medications. Given the relatively short half-lives of oral antipsychotics,^44,45^ patients are unlikely to have had antipsychotics in their system.

Another strength of the study was that there were no differences in BMI or subjective food ratings between patients with first-episode psychosis and controls. Although there were group differences in dietary intake of saturated fat, our findings remained unchanged when including BMI or saturated fat intake as covariates. Therefore, these factors are unlikely to be confounds. Although we included saturated fat intake as a second-level covariate, future studies should calculate the saturated fat content of food items and include these as first-level analysis covariates. In addition, while patients with first-episode psychosis were less likely to be employed or undertake weekly exercise, we have been unable to find literature indicating that these factors may moderate neural responses to food.

Although we investigated the effects of visual cues, these findings may not be generalizable to neural responses to olfactory or gustatory cues. Although the low-calorie condition consisted of relatively healthier food (eg, fruit and vegetables), they were not lower in carbohydrate or sugar content relative to the high-calorie condition, which may explain why the controls failed to show differences in functional activation in response to high-calorie cues vs low-calorie cues. Our study was powered to detect the effect size reported by Grimm et al.^24^ Moreover, our sample is the largest to date to investigate brain responses to food stimuli in psychosis, to our knowledge. However, we cannot exclude the possibility of type II error, and that smaller differences between patients and controls may have been undetected. However, the clinical significance of a smaller effect is unclear. A future study with a larger sample size is needed to exclude this possibility. Although we investigated patients in a fasted state, future studies could also investigate patients during fed states.

### Implications

Consistent with previous literature,^12,36,46^ patients and controls activated appetite-related neural networks including the striatum, insula, and orbitofrontal cortex in response to food cues. Although we found no evidence of striatal alterations in response to food cues in patients, paradigms involving the administration of monetary rewards have reported striatal alterations in patients with both treated and untreated schizophrenia.^18,19,20^ Thus, taken together with these prior findings, our findings indicate that patients with first-episode psychosis who are not taking antipsychotic medication do not show global impairments in striatal function. Instead, striatal dysfunction in individuals with schizophrenia may be specific to certain aspects of reward processing. This possibility warrants further testing in studies investigating multiple aspects of striatal function in the same patients.

Our finding that neural responses to food cues are unaltered early in the course of illness indicates that the neural mechanisms underlying subjective food preferences are not intrinsically dysregulated in individuals with schizophrenia. Taken with previous findings of altered striatal response to food cues in patients taking antipsychotics,^24,25^ our results indicate that antipsychotics may dysregulate neural processing of food cues, suggesting a mechanism for antipsychotic-associated weight gain. However, future longitudinal studies are needed to investigate this possibility.

Our finding that patients with first-episode psychosis who were not taking antipsychotic medication consume greater levels of saturated fat may be explained by differences in the homeostatic mechanisms involved in signaling satiety, such as peptide hormones involved in inhibiting food intake.^47^ Poor diets in patients may also be explained by differences in employment status between controls and patients with first-episode psychosis. In line with this finding, previous literature has shown that living in a low-income neighborhood is associated with the prevalence of obesity and poor dietary choices.^48^

There are shared genetic risk factors for schizophrenia and decreased BMI.^49^ Our finding that patients do not show an association between BMI and neural responses to food cues adds to the literature to suggest that the neural regulation of BMI may be altered in individuals with schizophrenia. However, further work is required to determine the direction of causality and the mechanisms involved.

Because making healthy dietary choices requires effective planning, future studies could also investigate whether negative symptom severity and/or cognitive deficits contribute to poor dietary choices in individuals with schizophrenia. Because unaffected first-degree relatives of patients with schizophrenia also show cardiometabolic alterations,^50^ future studies are also needed to disentangle the relative contributions of genetic and environmental factors for cardiometabolic dysfunction.

## Conclusions

We show for the first time, to our knowledge, that patients with first-episode psychosis who are not taking antipsychotic medication consume greater amounts of saturated fat relative to healthy controls but show normal neural responses to food cues in the fasted state. Although the neural mechanisms underlying subjective food preferences are not intrinsically dysregulated in individuals with schizophrenia, maladaptive eating patterns and alterations in the association between BMI and neural responses to food cues are established early in the course of schizophrenia.

## Notes

Supplement. **eAppendix.** Methods **eFigure 1.** Scatter Plot Showing the Association Between BMI and Mean Functional Activation in the Nucleus Accumbens in Response to Food vs Nonfood Cues in Healthy Controls **eFigure 2.** Scatter Plot Showing the Association Between BMI and Mean Functional Activation in the Nucleus Accumbens in Response to Food vs Nonfood Cues in Patients With First-Episode Psychosis
