# Supplementary material for: Neural Responsivity to Food Cues in Patients With Unmedicated First-Episode Psychosis
Source: JAMA Netw Open. 2019 Jan 11;2(1):e186893. doi: 10.1001/jamanetworkopen.2018.6893 (PMC6420094; doi:10.1001/jamanetworkopen.2018.6893)
Supplement: Supplement. — eAppendix. Methods eFigure 1. Scatter Plot Showing the Association Between BMI and Mean Functional Activation in the Nucleus Accumbens in Response to Food vs Nonfood Cues in Healthy Controls eFigure 2. Scatter Plot Showing the Association Between BMI and Mean Functional Activation in the Nucleus Accumbens in Response to Food vs Nonfood Cues in Patients With First-Episode Psychosis [file jamanetwopen-2-e186893-s001.pdf]

Supplementary Online Content

Borgan F, O'Daly O, Hoang K, et al. Neural responsivity to food cues in patients with unmedicated first-episode psychosis. *JAMA Netw Open.* 2019;2(1):e186893. doi:10.1001/jamanetworkopen.2018.6893

eAppendix. Methods
eFigure 1. Scatter Plot Showing the Association Between BMI and Mean Functional Activation in the Nucleus Accumbens in Response to Food vs Nonfood Cues in Healthy Controls
eFigure 2. Scatter Plot Showing the Association Between BMI and Mean Functional Activation in the Nucleus Accumbens in Response to Food vs Nonfood Cues in Patients With First-Episode Psychosis

This supplementary material has been provided by the authors to give readers additional information about their work.

© 2019 Borgan F et al. *JAMA Network Open.*

eAppendix. Methods

Food cue fMRI paradigm

All colour photographs were previously validated and standardized for size, complexity and resolution so that they were equal across conditions [38]. The task consisted of 16 blocks, divided into 4 epochs which were repeated 4 times in a pseudorandom order. Each epoch consisted of the presentation of 1 rest block and 3 task blocks corresponding to each of the conditions described above. Each rest block consisted of the presentation of a fixation cross for 24 seconds. Each task block involved the consecutive presentation of 8 photographs for 3 seconds each. At the end of each task block, volunteers were asked to rate how pleasant the images were, using a 9-point Likert visual analogue scale (VAS) over 7.5 seconds. Volunteers responded to the VAS by moving a cursor to the left or right by pressing the respective left and right buttons using a 2-button button box. The duration of each task block was 31.5 seconds. The duration of each epoch was 118.5 seconds. Images were projected through the radiofrequency (RF) window onto a screen at the bottom of the scanner. The total task duration was 9:06 minutes.

© 2019 Borgan F et al. *JAMA Network Open.*

eFigure 1. Scatter Plot Showing the Association Between BMI and Mean Functional Activation in the Nucleus Accumbens in Response to Food vs Nonfood Cues in Healthy Controls

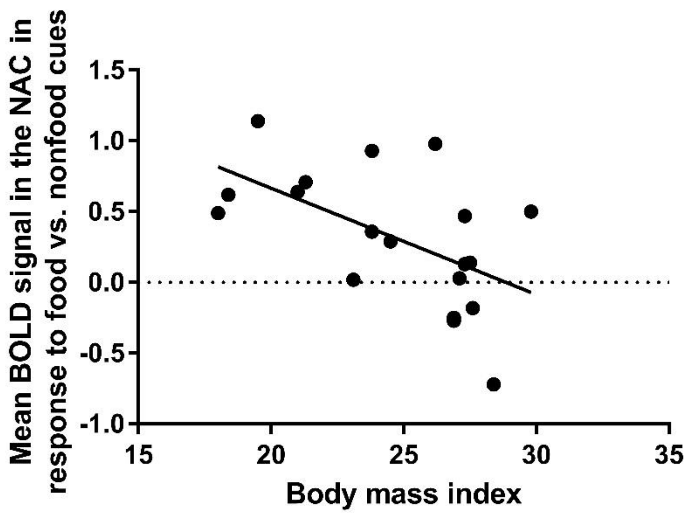

© 2019 Borgan F et al. *JAMA Network Open.*

eFigure 2. Scatter Plot Showing the Association Between BMI and Mean Functional Activation in the Nucleus Accumbens in Response to Food vs Nonfood Cues in Patients With First-Episode Psychosis

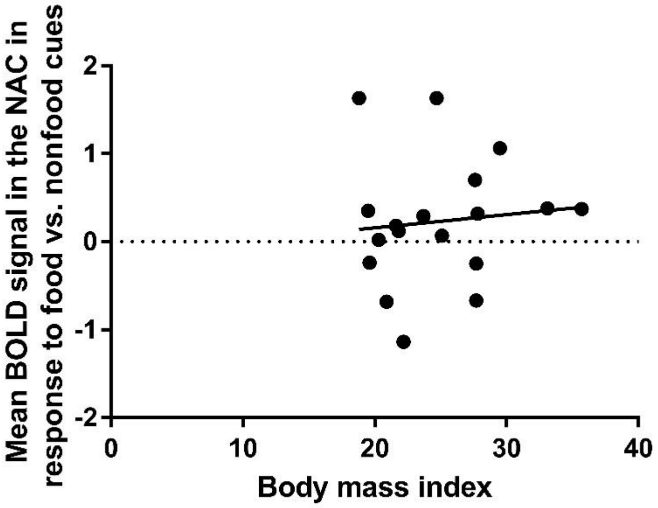

© 2019 Borgan F et al. *JAMA Network Open.*
